# Molecular vs. endoscopic sexing of domestic pigeons (*Columba livia domestica*): accuracy, sample type, and practical recommendations for bird sexing

**DOI:** 10.3389/fvets.2026.1936668

**Published:** 2026-08-26

**Authors:** Maria-Carmen Turcu, Alessandro Vetere, Anamaria Ioana Pastiu, Michela Ablondi, Lucia Victoria Bel, Dana Liana Pusta

**Affiliations:** 1 Department of Genetics and Hereditary Diseases, Faculty of Veterinary Medicine, University of Agricultural Sciences and Veterinary Medicine Cluj-Napoca, Cluj-Napoca, Romania; 2 New Companion Animals Clinic, Department of Surgery and Intensive Care, Faculty of Veterinary Medicine, University of Agricultural Sciences and Veterinary Medicine Cluj-Napoca, Cluj-Napoca, Romania; 3 Department of Veterinary Science, University of Parma, Parma, Italy

**Keywords:** celioscopy, CHD1W and CHD1Z, domestic pigeon, endoscopic sexing, minimally invasive sexing, molecular sexing, PCR

## Abstract

**Introduction:**

The domestic pigeon (*Columba livia domestica*) lacks distinct sexual dimorphism, making sex determination based on external morphology unreliable. This species can serve as a model for evaluating avian sex determination methods and identifying minimally invasive and effective techniques for monomorphic birds. This study aimed to compare several sexing approaches and assess their reliability.

**Methods:**

Twenty-five deceased pigeons were examined. Sex was initially determined by celioscopy, followed by necropsy and direct visualization of the gonads. During necropsy, feather samples, oral swabs, and blood clots were collected. DNA was extracted from each sample type, and polymerase chain reaction (PCR) amplification was performed for molecular sex determination.

**Results:**

Molecular sexing correctly determined sex in 100% of cases (95% CI: 86.7%–100.0%), whereas necropsy accurately identified sex in 84.0% (95% CI: 65.3%–93.6%) of cases and celioscopy in 72.0% (95% CI: 52.4%–85.7%). Juvenile pigeons with small or underdeveloped gonads could only be accurately sexed using molecular methods. Among the sample types, blood clots showed the highest amplification success (100%; 95% CI: 86.7%–100.0%) and DNA concentration (181.68 ± 138.83 ng/μL), followed by oral swabs (88.0%; 95% CI: 70.0%–95.8%; 84.69 ± 79.85 ng/μL) and feathers (80.0%; 95% CI: 60.9%–91.1%; 9.54 ± 11.07 ng/μL).

**Discussion:**

Blood clot samples provided the highest success rate for molecular sexing; however, oral swabs represent a practical and minimally invasive alternative. Molecular sexing using oral swabs is therefore a reliable method for sex determination in pigeons, including newly hatched chicks, and represents a valuable tool for breeders and avian practitioners.

## 1 Introduction

Accurate sex determination is essential in avian medicine, breeding management, and wildlife conservation. However, more than half of bird species lack distinct sexual dimorphic characteristics, making visual sex identification unreliable (1, 2). This limitation is particularly evident in domestic pigeons (*Columba livia domestica*), in which males and females exhibit only subtle morphological differences (2). As a result, determining sex based solely on external characteristics may lead to misidentification, especially in juvenile birds.

Traditional methods of avian sex determination are primarily based on secondary sexual characteristics such as body size, head shape, plumage patterns, or behavioral traits. In some species, cloacal inspection or vent sexing may also be used to evaluate anatomical differences within the cloaca. Behavioral observations, including courtship displays, territorial behavior, and mating rituals, may provide additional indications of sex. However, these approaches are often tardive, time-consuming, require prolonged observation, and may remain inconclusive in monomorphic species (3, 4).

Surgical techniques, including celiotomy and celioscopy, allow direct visualization of the gonads and therefore provide highly accurate sex determination. Endoscopic sexing involves the insertion of an endoscope into the celomic cavity under general anesthesia, enabling identification of testes or ovaries. Although reliable, these procedures are invasive, require specialized equipment and trained personnel, and involve anesthetic risks that limit their routine application in clinical or breeding settings (3, 5).

Molecular sexing has become the preferred method for avian sex determination due to its high accuracy, reproducibility, and minimal invasiveness (6–11). In birds, males possess two Z chromosomes (ZZ), whereas females possess one Z and one W chromosome (ZW). Molecular techniques targeting the chromo-helicase-DNA binding (CHD1) gene allow differentiation between these chromosomes through polymerase chain reaction (PCR) amplification. The presence of both CHD1Z and CHD1W fragments indicates a female, while the presence of a single CHD1Z fragment indicates a male (1, 11). PCR-based sexing has demonstrated accuracy rates exceeding 99% and can be performed using a variety of biological samples, including blood, feathers, and buccal or oral swabs (12–17).

Domestic pigeons are widely distributed and commonly used in breeding, racing, and research contexts. Columbiformes are typically monogamous and exhibit strong pair bonding and biparental care, making accurate sex identification particularly important for breeding management (2). Because pigeons lack pronounced sexual dimorphism yet are readily available for study, they represent a suitable model species for evaluating different avian sex determination techniques.

The primary objective of this study was to perform a comparative evaluation of endoscopic, necropsy, and molecular sexing methods in domestic pigeons to assess their diagnostic performance and practical applicability. Unlike previous studies, we simultaneously compared three biological sample types (blood clots, oral swabs, and feathers) collected from the same individuals for molecular sex determination, while also evaluating the performance of endoscopic sexing when performed by a novice operator and validated by necropsy findings. Particular emphasis was placed on the applicability of these methods in juvenile pigeons, in which gonadal differentiation is often incomplete, and on providing practical recommendations for pigeon breeding and avian clinical practice.

## 2 Material and method

### 2.1 Study animals

This study included only domestic pigeon (*Columba livia domestica*) cadavers admitted in 2023 to the New Companion Animals Veterinary Clinic, Faculty of Veterinary Medicine, University of Agricultural Sciences and Veterinary Medicine, Cluj-Napoca, Romania. To ensure consistency, quality, and feasibility while allowing meaningful comparisons across methods, only birds with complete datasets were included in the current study. The pigeons were either naturally deceased or euthanized due to severe, life-threatening conditions unrelated to this study. All individuals lacked clear sexual dimorphic traits. The cohort comprised different age groups based on Tudor's classification (2): 18 adults (>6 months), 6 juveniles (<6 months), and 1 newly hatched pigeon (approximately 7 days old).

**Scheme 1:**
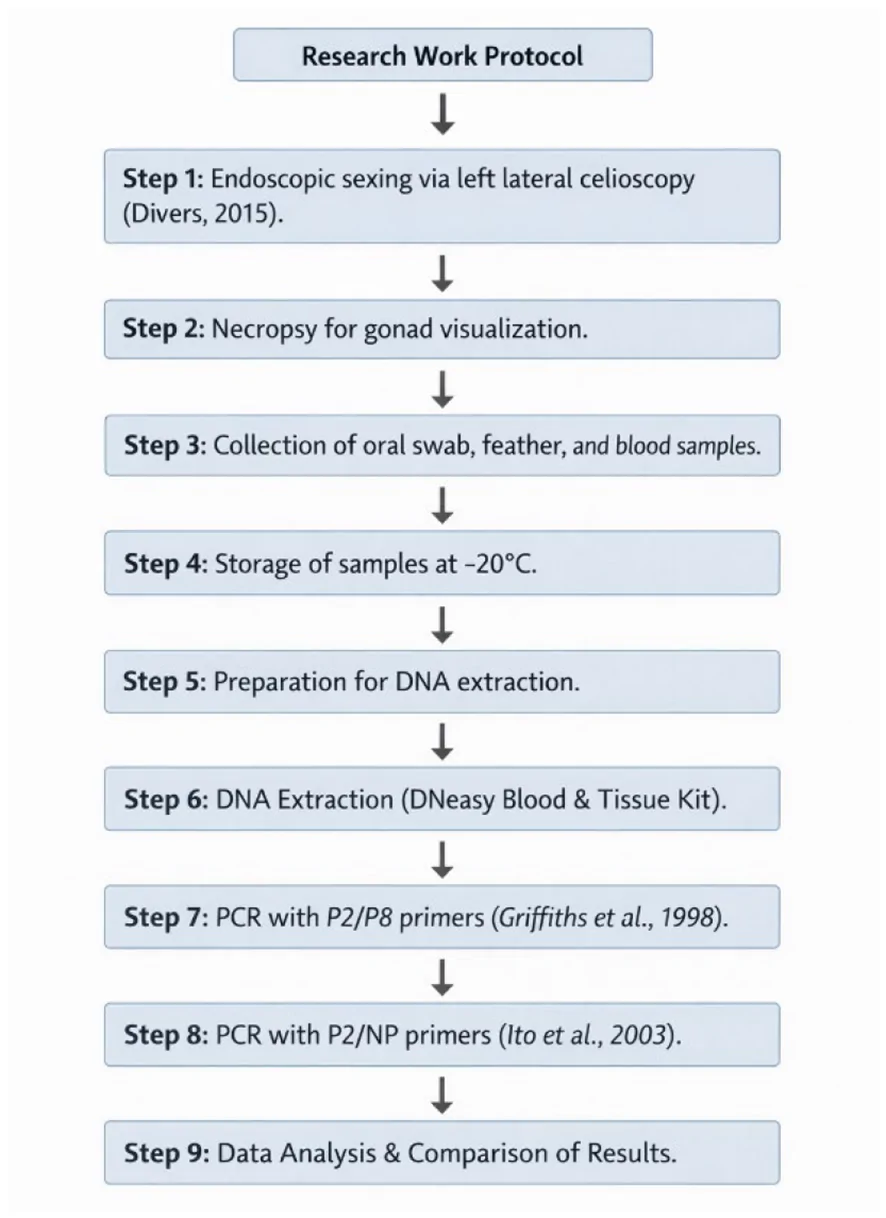
Flowchart of the research protocol for the comparative study of sex determination techniques in domestic pigeons. Source: Original work by the authors, created with the assistance of ChatGPT.

The current study was conducted according to the workflow presented in Scheme 1.

### 2.2 Endoscopic sexing

**Figure 1:**
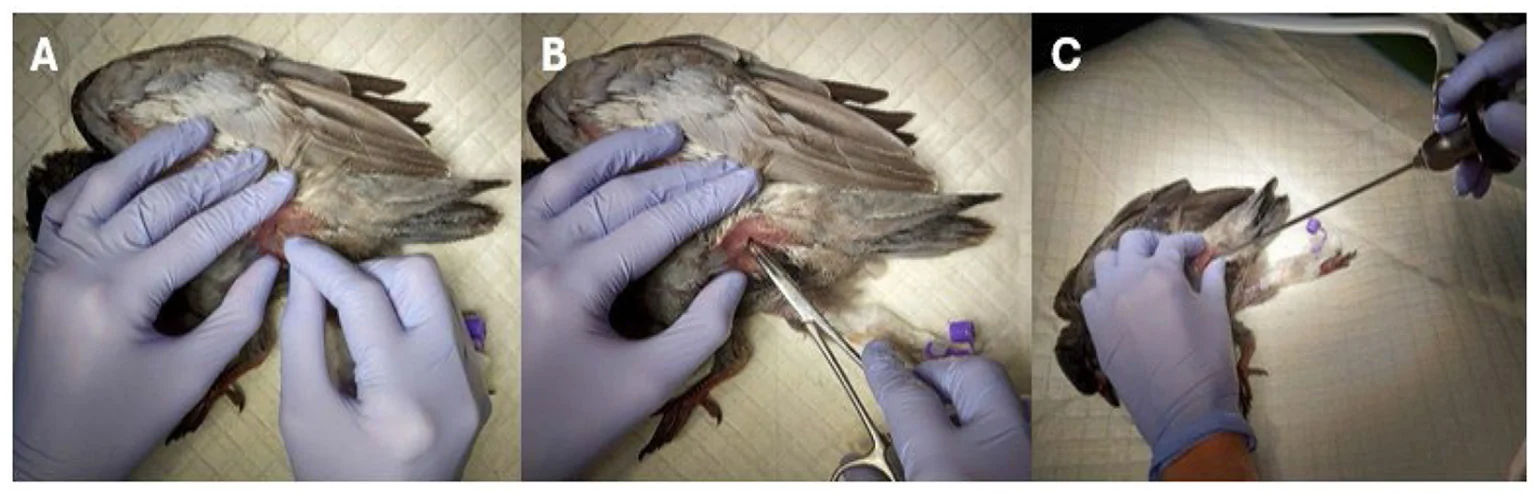
**(A)**–**(C)** Illustrate celioscopic access through the abdominal wall and the insertion of a 2.7 mm telescope. **(A)** Shows the initial skin incision. **(B)** Demonstrates the careful advancement of curved Pean forceps through the abdominal muscles to create a small opening in the muscle layers. **(C)** Presents the perpendicular insertion of the 2.7 mm telescope through this opening to allow endoscopic examination.

Endoscopic sexing was performed on all pigeon carcasses (*n* = 25) using celioscopy *via* left lateral approach as described by Divers (18). The pigeons were positioned in right lateral recumbency with the wings extended dorsally. The left limb was pulled cranially while the right limb remained in a neutral position. The target area was defined by anatomical landmarks forming a triangular region bounded cranially by the last rib, caudally by the medial crural flexor muscle, and ventrally by a line passing through the sternum. Feathers from the left flank were plucked and the skin was disinfected. A 2–3 mm incision was made caudal to the last rib and ventral to the medial crural flexor muscle. The caudal thoracic air sac was accessed using curved Pean forceps that created a small tearing in the muscle layers. A 2.7 mm telescope (Karl Storz Veterinary Endoscopy America Inc., USA) was then inserted perpendicularly to the animal into the caudal thoracic air sac (Figure 1). Successful penetration of the air sac was confirmed by visualization of anatomical structures including the left lung, cranial thoracic air sac, abdominal air sac, liver, proventriculus, and ribs with intercostal muscles. The telescope was then directed caudally to access the left abdominal air sac by perforating the air sac membrane. The kidney–gonad–adrenal gland triad was identified, with the gonads located at the cranial pole of the kidney. In sexually mature pigeons, enlarged testicles could partially obscure the adrenal gland (18).

### 2.3 Necropsy and gonad identification

Following endoscopic examination, necropsy was performed on all carcasses to confirm sex through direct visualization of the gonads. The birds were positioned in dorsal recumbency. Access to the celomic cavity was obtained through a midline incision extending from the caudal end of the sternum to the cloaca. Two additional incisions were made along the hypochondrial margins toward the ilium. The ribs were sectioned up to the scapular girdle (scapula, coracoid, and clavicle), allowing elevation of the sternum and pectoral muscles (19). After opening the celomic cavity, the gastrointestinal tract was gently displaced to expose the kidneys. The gonads were identified at the cranial pole of the kidneys and visually examined to determine sex.

### 2.4 Sample collection

During necropsy, three types of biological samples were collected from each individual (*n* = 25), feathers, oral swabs, and blood clots (Table 1). Blood clots were collected from the cardiac ventricles and placed in sterile 1.5 ml Eppendorf tubes. Primary feathers (2–4 feathers with intact calamus) were plucked from the wings and stored in labeled plastic bags. Down feathers were collected from the newly hatched chick. Oral samples were obtained using sterile cotton swabs (Prima, Taizhou Honod Medical Co., Ltd., Zhejiang, China), following the protocol described by Handel et al. (20). At least two swabs were collected per individual. All samples were collected using individual sterile gloves, labeled, and stored at −20 °C until DNA extraction. Duplicate samples were also stored under identical conditions.

**Table 1:** DNA samples included in this study.

Species	Age	No. of cadavers	Oral swabs	Feathers	Blood clots
Domestic pigeon (*Columba livia domestica*)	adults	18	18	18	18
	juveniles	6	6	6	6
	newly hatched	1	1	1	1
Total	-	25	25	25	25

### 2.5 DNA extraction and polymerase chain reaction (PCR)

Genomic DNA extraction was conducted on all collected samples (*n* = 75) from pigeons, encompassing feathers (*n* = 25), oral swabs (*n* = 25), and blood clots (*n* = 25). The DNeasy Blood and Tissue kit (Qiagen, Hilden, Germany) was used for DNA extraction, following the manufacturer's protocol. Blood clots were initially collected in 1.5 ml sterile Eppendorf tubes, while oral swabs were transferred to 1.5 ml Eppendorf tubes using sterile scissors. Feathers underwent calamus sectioning into small 2–3 mm pieces *via* a sterile scalpel. Subsequently, feathers were mechanically disrupted through high-speed shaking with steel beads using TissueLyserII (Qiagen, US) (21). DNA extraction encompassed blood clots, 25 mg of feather, and the entire swab.

The quantity and purity of the extracted DNA were assessed by spectrophotometry using a NanoDrop ND-1000 (NanoDrop Technologies, Inc., Rockland, ME, USA). DNA concentration was measured in ng/μl, and 5 μl of each DNA sample was analyzed at absorbance ratios of 260/280 and 260/230 nm. These measurements were used to evaluate DNA purity and to allow comparison of DNA quantity and quality between the different sample types.

The extracted DNA underwent further testing for the presence of specific genes CHD1W and CHD1Z through two PCR protocols, with the PCR reaction mixture comprising 25 μl and including 12.5 μl of MyTaq Red HS Mix (Meridian Bioscience, USA) and 25 pM of each primer, while the DNA template volume was at least 50 ng/ul/reaction.

The DNA was tested using two PCR protocols to identify the CHD1W and CHD1Z genes.

**PCR protocol 1**. The identification of the CHD1 gene was performed according to the protocol described by Griffiths et al. (1), using the primers P2 (5′-TCT GCA TCG CTA AAT CCT TT-3′) and P8 (5′-CTC CCA AGG ATG AGR AAY TG-3′; Generi-Biotech, Hradec Králove, Czech Republic). Cycling conditions were: 95 °C for 5 min for initial denaturation, followed by 48° C for 45 s, 72 °C for 45 s and 95 °C for 30 s (35 cycles); 48 °C for 1 min and final extension at 72 °C for 5 min.

**PCR protocol 2**. The identification of the CHD1 gene was performed according to the protocol described by Ito et al. (22), using the primers P2 (5′-TCT GCA TCG CTA AAT CCT TT-3′) and NP (5′-GAG AAA CTG TGC AAA ACAG-3′; Generi-Biotech, Hradec Králove, Czech Republic). Cycling conditions were: 95 °C for 5 min for initial denaturation, followed by 52 °C for 45 s, 72 °C for 45 s and 95 °C for 30 s (35 cycles); 50 °C for 1 min and final extension at 72 °C for 5 min.

Aliquots of each PCR product were electrophoresed on 3% agarose gel stained with SYBR^®^ Safe DNA gel stain (Invitrogen, Carlsbad, California), and examined for the presence of the specific fragment under UV light (Bio-Rad BioDoc-ItTM Imagine System). DNA fragment size was compared with a standard molecular weight, 100 bp DNA ladder (Fermentas; Thermo Fisher Scientific, Waltham, Massachusetts).

### 2.6 Statistical analysis

Statistical analyses were conducted to evaluate the agreement between Endoscopy, Necropsy, and Molecular sexing methods in determining the sex of domestic pigeons. The primary aim was to assess the accuracy and consistency of each sexing technique and to examine the level of agreement between them. To achieve this, two statistical tests were employed.

First, Cohen's Kappa was used to quantify the degree of agreement between pairs of methods: Endoscopy vs. Molecular and Necropsy vs. Molecular. Cohen's Kappa accounts for the agreement that could occur by chance, providing a measure of the true concordance between the methods. The kappa value ranges from −1 (complete disagreement) to 1 (perfect agreement), with values closer to 1 indicating a stronger agreement between the methods.

Second, McNemar's Test was applied to assess whether there was any statistically significant discordance (i.e., misclassification or disagreement) between the two methods being compared. This test is particularly useful for evaluating paired nominal data and determining if there is a significant difference in the classifications provided by the two methods.

Contingency tables were created for each comparison. For the Endoscopy vs. Molecular comparison, seven cases where Endoscopy returned an “Undetermined” result were excluded. Similarly, for the Necropsy vs. Molecular comparison, four cases where Necropsy returned “Undetermined” were excluded. These exclusions ensured that only cases with definitive results were included in the statistical tests, providing a more reliable comparison of the methods.

In addition, DNA quantity obtained from the samples was analyzed to evaluate sample quality. Descriptive statistical parameters, including the mean and standard deviation, were calculated and compared between sample groups.

All statistical analyses were conducted using the Statsmodels Python library (https://www.statsmodels.org/stable/index.html, accessed 02/01/2025). Cohen's Kappa was calculated using the cohens_kappa function, while McNemar's test was performed using the mcnemar function. A significance threshold of *p* < 0.05 was applied to all tests.

DNA concentration and spectrophotometric purity (A260/A280 and A260/A230 ratios) were summarized using descriptive statistics and are presented as mean ± standard deviation (SD). Differences in DNA concentration among blood clots, oral swabs, and feather samples were evaluated using the Kruskal-Wallis test. When a significant overall difference was detected, pairwise comparisons were performed using the Mann-Whitney U test with Bonferroni correction for multiple comparisons.

## 3 Results

### 3.1 Endoscopic sexing

**Figure 2:**
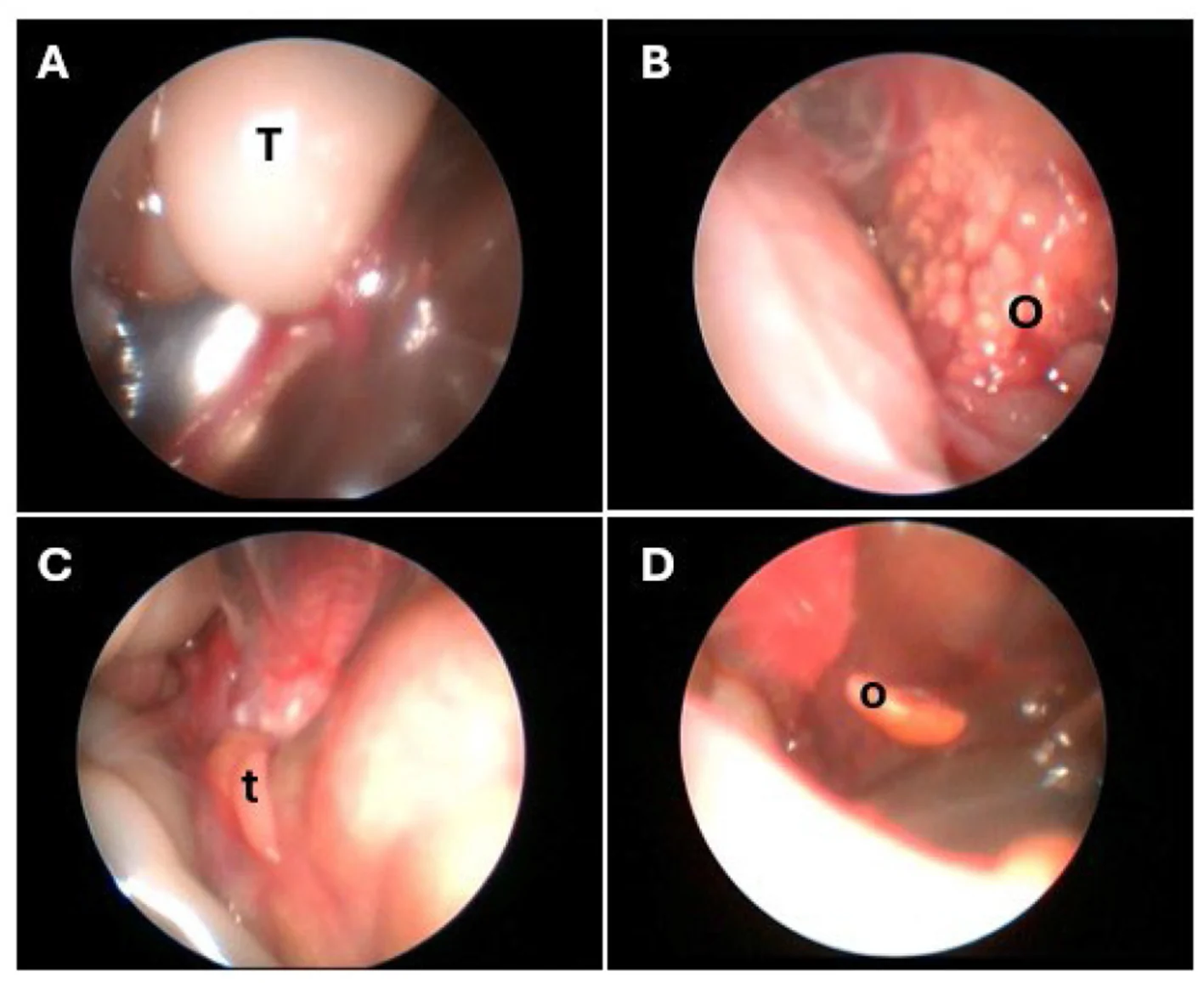
Celioscopic visualization of the gonads in a domestic pigeon: **A**-mature testis (T); **B**-mature ovary (O); **C**-immature testis (t); **D**-immature ovary (o).

Endoscopic examination *via* left lateral celioscopy allowed visualization of the gonads located at the cranial pole of the kidneys, forming the kidney–gonad–adrenal gland triad. Among the 25 examined pigeons, endoscopy identified 14 males and 4 females, while sex determination was not possible in seven individuals. In adult pigeons, mature testes appeared oval, smooth, and highly vascularized (Figure 2A). In some cases, the contralateral (right) testicle was visible through the abdominal air sac. Mature ovaries showed a characteristic cluster-like appearance with multiple follicles at different stages of development (Figure 2B). In juvenile pigeons, immature testes were smooth and kidney bean-shaped (Figure 2C), whereas immature ovaries were irregular and exhibited shallow grooves with poorly defined follicles (Figure 2D). Sex determination in juveniles was more challenging due to the small size and limited differentiation of the gonads.

### 3.2 Necropsy findings

**Figure 3:**
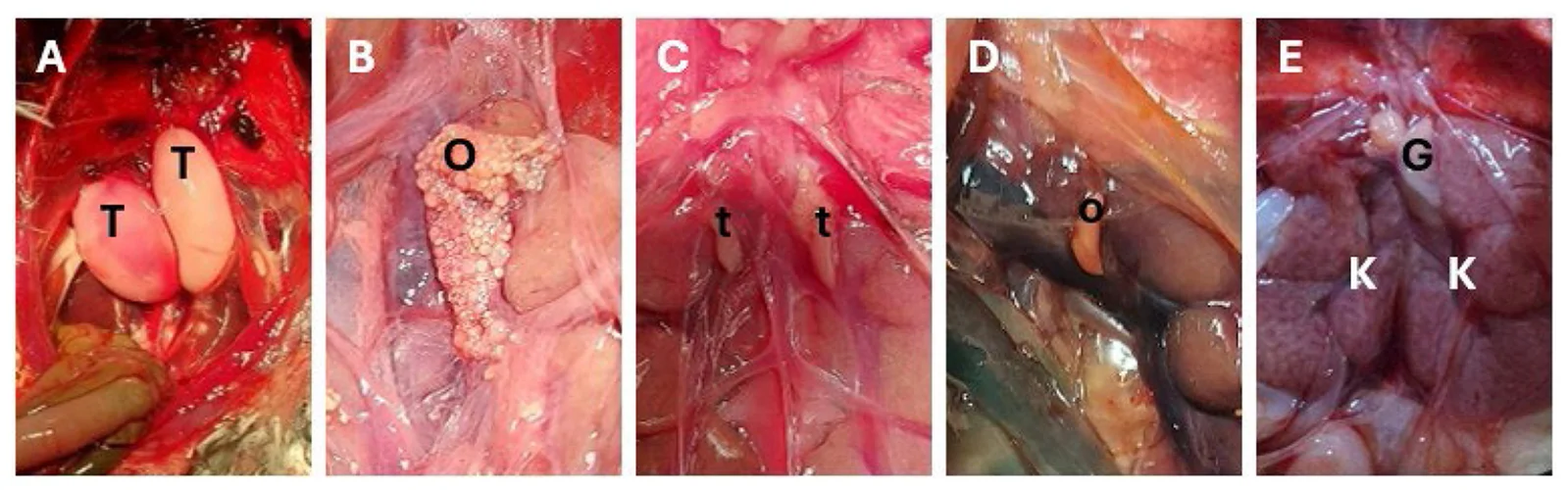
Necropsy identification of gonads in domestic pigeons: **A**: mature testicles (T); **B**: mature ovary (O); **C**: two immature testes (t); **D**: single immature ovary (o); **E**: kidneys (K) and poorly differentiated gonads (G) in a newly hatched pigeon.

Necropsy examination allowed direct visualization of the gonads in the celomic cavity. Of the 25 examined carcasses, necropsy identified 15 males and 6 females, while sex determination remained inconclusive in four individuals. In adult pigeons, mature testes were oval with a smooth surface and prominent vascularization (Figure 3A). The mature ovary presented a grape-like structure composed of multiple follicles at different developmental stages (Figure 3B). In juvenile pigeons, immature testes appeared elongated and smooth (Figure 3C), whereas immature ovaries displayed irregular surfaces with poorly differentiated follicles (Figure 3D). In the newly hatched chick, the gonads were extremely small and poorly differentiated, making visual identification difficult (Figure 3E).

### 3.3 Molecular sexing

Molecular sexing was performed on all 25 pigeons using three types of biological samples: blood clots, oral swabs, and feathers. The DNeasy Blood and Tissue Kit (Qiagen, Hilden, Germany) yielded high-quality and quantity of DNA, demonstrating its suitability for reliable DNA extraction for molecular sexing in pigeons.

**Figure 4:**
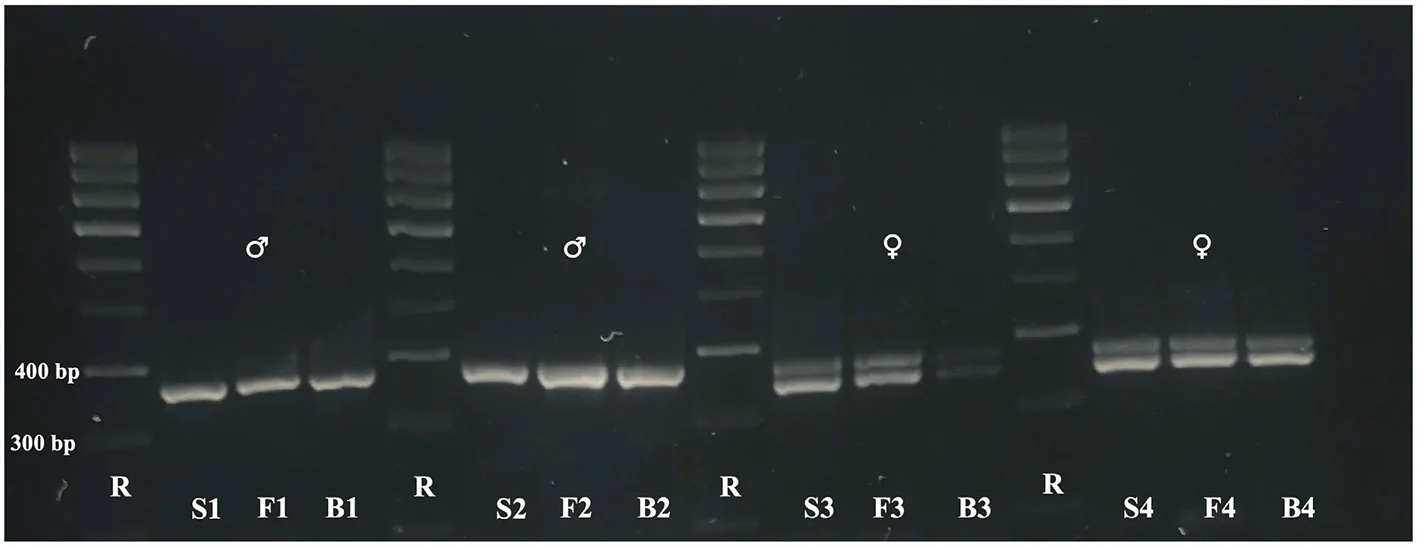
DNA sexing of domestic pigeons using PCR with P2/P8 primers (1). Males (♂) show a single CHD1Z band, whereas females (♀) display two bands corresponding to CHD1Z and CHD1W. Legend: R—corespond to size standard (100-bp DNA ladder); S—oral swab samples; F—feather samples; B—blood clot samples; 1—pigeon no. 5; 2—pigeon no. 7; 3—pigeon no. 15; 3—pigeon no. 19.

**Figure 5:**
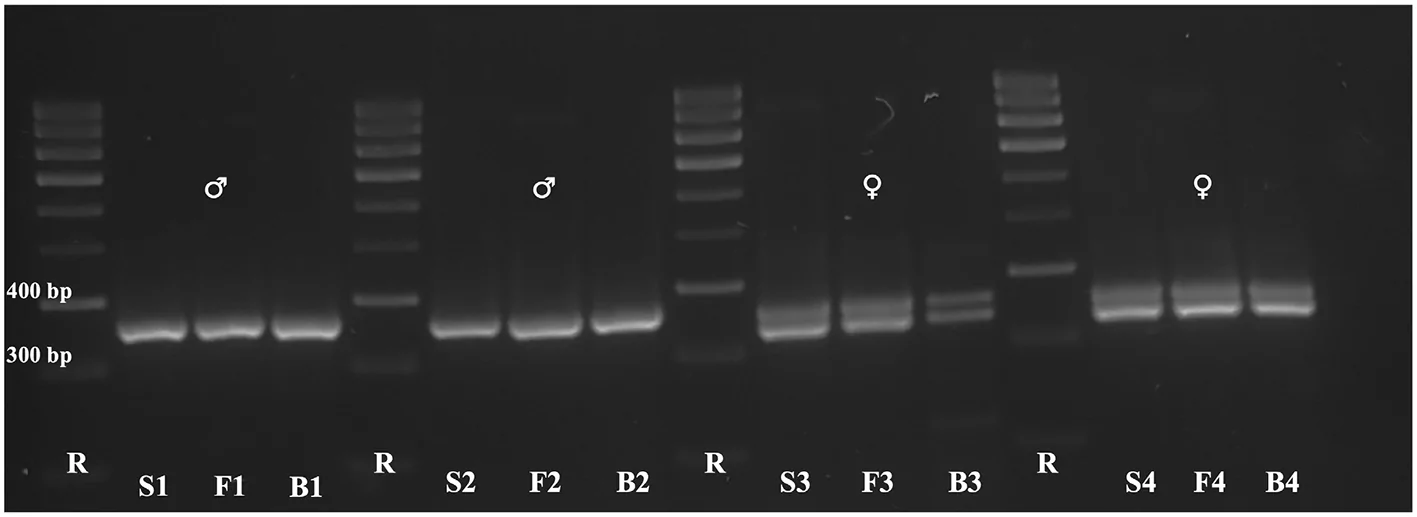
DNA sexing by PCR with primers P2/NP (22). Males (♂) show a single CHD1Z band, whereas females (♀) display two bands corresponding to CHD1Z and CHD1W from samples of oral swabs, feathers and blood clots. Legend: R—corespond to size standard (100-bp DNA ladder); S—oral swab samples; F—feather samples; B—blood clot samples; 1—pigeon no. 5; 2—pigeon no. 7; 3—pigeon no. 15; 3—pigeon no. 19.

PCR amplification of the CHD1 gene produced distinct banding patterns for males and females. Male individuals displayed a single band corresponding to the CHD1Z gene, whereas females exhibited two bands corresponding to the CHD1Z and CHD1W genes. DNA amplification was performed using two PCR protocols using the P2/P8 primers (1) and the P2/NP primers (22) (Figures 4 and 5). The P2/NP protocol offered sharper, clearer and more distinct amplification bands, indicating its superior clarity and making it the recommended method by the authors for molecular sexing.

**Figure 6:**
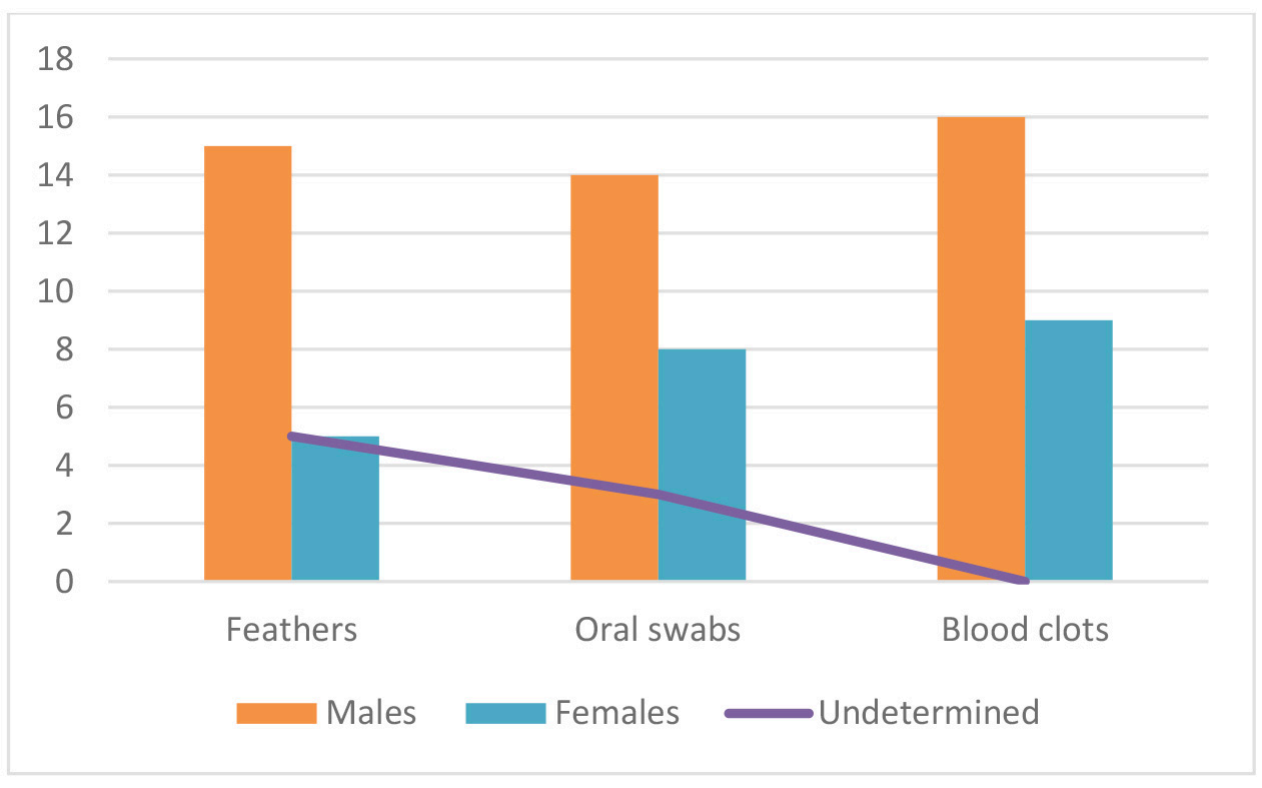
Results of DNA sexing in domestic pigeon carcasses using oral swabs, feathers and blood clots samples.

Molecular analysis identified 16 males and 9 females, with no indeterminate results. Among the biological sample types tested, blood clot samples had successful amplification in 100.0% (95% CI: 86.7%−100.0%) of cases. Oral swabs achieved successful amplification in 88.0% (95% CI: 70.0%−95.8%) of cases, while feathers achieved successful amplification in 80.0% (95% CI: 60.9%−91.1%) of cases (Figure 6).

Spectrophotometric analysis revealed clear differences in DNA quantity among sample types (*H* = 39.60, *p* < 0.0001). Blood samples showed the highest mean DNA concentration (191.16 ± 136.30 ng/μl), followed by oral swabs (87.27 ± 83.16 ng/μl), while feathers exhibited substantially lower values (13.72 ± 17.85 ng/μl). Pairwise comparisons revealed that blood clot samples yielded significantly higher DNA concentrations than both oral swab (*p* = 0.0044) and feather samples (*p* < 0.0001). Furthermore, DNA concentrations obtained from oral swabs were significantly higher than those obtained from feathers (*p* < 0.0001). Spectrophotometric analysis revealed differences not only in DNA concentration but also in DNA purity (Table 2). Blood clot samples showed A260/A280 ratios closest to the expected value for pure DNA, whereas oral swab and feather samples exhibited slightly higher A260/A280 ratios (Table 2).

**Table 2:** Spectrophotometric analysis of DNA from blood clot, oral swab, and feather samples; Only samples with valid spectrophotometric measurements were included in the calculations.

Sample type	Spectrophotometric analysis	
	**DNA concentration (ng/μL), mean** **±SD (range)**	**A260/A280, mean** **±SD (range)**	**A260/A230, mean** **±SD (range)**
Blood clots (*n* = 17)	191.16 ± 136.30 (39.9–492.5)	1.80 ± 0.13 (1.50–1.91)	2.00 ± 0.22 (1.57–2.34)
Oral swabs (*n* = 21)	87.27 ± 83.16 (26.8–350.7)	2.11 ± 0.27 (1.86–2.78)	2.23 ± 0.38 (1.84–2.95)
Feathers (*n* = 23)	13.72 ± 17.85 (0.6–70.7)	2.10 ± 0.39 (1.61–2.80)	1.65 ± 0.25 (1.28–2.11)

The observed range (minimum-maximum) shown in parentheses.

**Figure 7:**
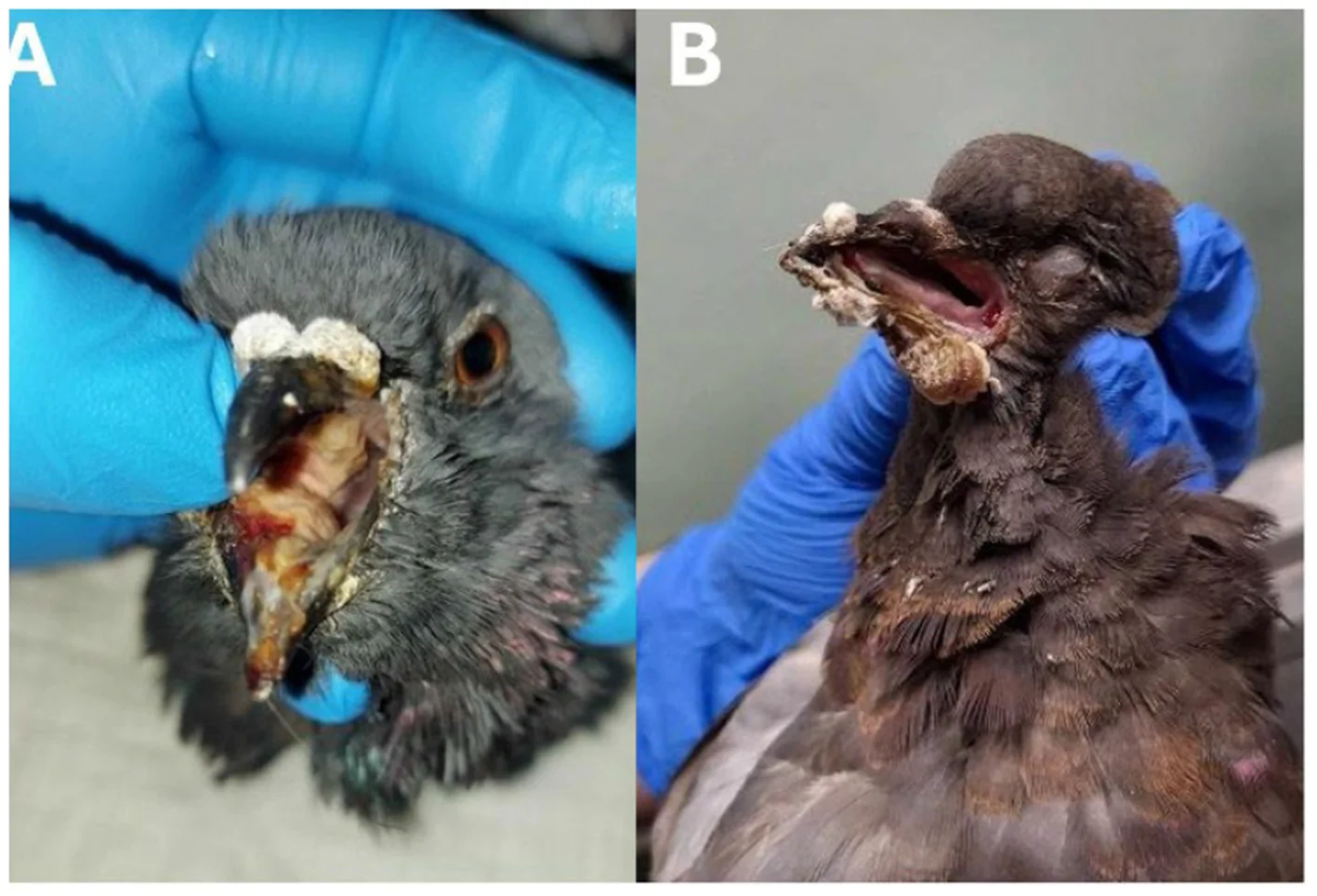
Necropsy macroscopic view of oral pseudomembranes and necrotic oral lesions in two domestic pigeons included in this study, with suspected poxvirus infection, contributing to reduced DNA results from oral swabs.

In pigeons affected by avian poxvirus with buccal lesions, oral swab samples obtained measurable DNA concentrations ranging from 44.5 to 70.7 ng/μl, exceeding the mean values obtained from feather samples (9.54 ± 11.07 ng/μl). This finding highlights the reduced DNA quantity and detectability associated with feather-derived material compared with both blood and oral swab samples. Oral swab-based molecular sexing achieved 88% (95% CI: 70.0%−95.8%) accuracy. Reduced accuracy was associated with two pigeons presenting oral lesions consistent with avian poxvirus infection, which showed lower DNA concentrations (44.5–70.7 ng/μl) compared with the overall mean for oral swabs (84.69 ± 79.85 ng/μl). These lesions, characterized by pseudomembranous and necrotic material, likely interfered with DNA collection, extraction, and amplification (Figure 7).

### 3.4 Statistical analysis

A total of 25 domestic pigeon carcasses were evaluated using three sex determination methods: endoscopy, necropsy, and molecular sexing. Molecular sexing successfully determined the sex of all individuals (100%; 95% CI: 86.7%−100.0%), identifying 16 males and 9 females with no indeterminate results. Necropsy allowed sex determination in 21 individuals (84%; 95% CI: 65.3%−93.6%), identifying 15 males and 6 females, while 4 individuals remained undetermined. Endoscopic examination provided definitive results in 18 individuals (72%; 95% CI: 52.4%−85.7%), identifying 14 males and 4 females, with 7 cases classified as undetermined. The comparative results of the three methods are summarized in Table 3. Molecular sexing demonstrated the highest diagnostic efficiency, followed by necropsy and endoscopic sexing. Juvenile pigeons with poorly differentiated gonads could only be reliably sexed using molecular techniques.

**Table 3:** Sexing results of the 25 domestic pigeons using celioscopy, necropsy, and molecular method.

Domestic pigeons (*Columba livia domestica*)	Sexing methods		
	**Endoscopy**	**Necropsy**	**PCR**
Males	14	15	16
Females	4	6	9
*Undetermined sex*	*7*	*4*	*0*
Total	25	25	25
Efficiency of method	72%	84%	100%
95% Confidence Intervals (Wilson method)	52.4–85.7%	65.3–93.6%	86.7–100.0%

Agreement between the sex determination methods was evaluated using Cohen's Kappa and McNemar's test.

Endoscopy vs. molecular sexing: the results of the statistical analyses revealed a perfect agreement between Endoscopy and Molecular sexing methods. The Cohen's Kappa value for this comparison was 1.0, indicating that the classifications made by Endoscopy were identical to those provided by Molecular sexing for the cases where definitive results were obtained. Additionally, the McNemar *p*-value for this comparison was 1.0, which means there was no statistically significant disagreement between the two methods. This result suggests that, when Endoscopy provided clear sexing results, they were consistent with the results from Molecular sexing.

Necropsy vs. molecular sexing: Similarly, the comparison between Necropsy and Molecular sexing also demonstrated perfect agreement. Cohen's Kappa value for this comparison was 1.0, indicating that Necropsy results were in perfect alignment with the Molecular sexing outcomes for the cases where definitive results were obtained. The McNemar *p*-value for this comparison was also 1.0, showing that there was no significant discordance between the two methods. This further supports the conclusion that Necropsy, when performed correctly, provides results consistent with Molecular sexing.

Both Cohen's Kappa and McNemar's Test indicated that Endoscopy and Necropsy methods exhibit perfect agreement with Molecular sexing. The findings suggest that, for pigeons where definitive sexing results were obtained *via* Endoscopy or Necropsy, these methods are in full agreement with the results derived from Molecular sexing. Furthermore, the McNemar test showed no significant disagreement between the methods, reinforcing the reliability and consistency of Endoscopy and Necropsy as sexing techniques in domestic pigeons.

**Figure 8:**
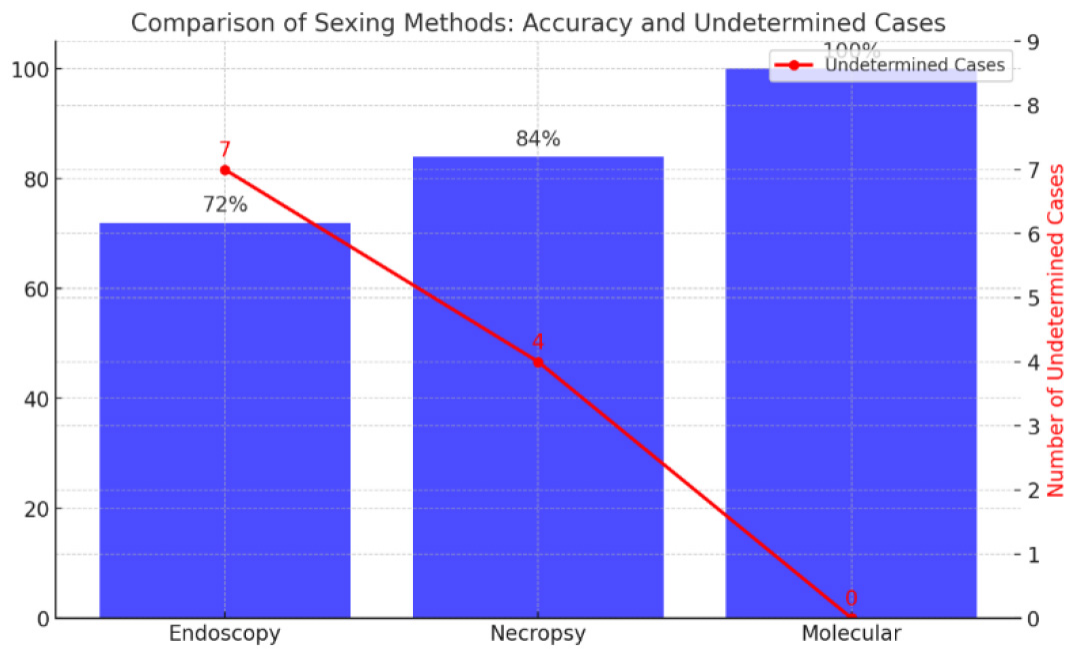
Comparison of the three sexing methods—Endoscopy, Necropsy, and Molecular sexing—in terms of accuracy (%) (blue bars) and the number of undetermined cases (red line). Molecular sexing achieved 100% accuracy with zero undetermined cases, while endoscopy and necropsy had lower accuracy (72 and 84%, respectively) and higher numbers of undetermined cases (7 and 4, respectively). The chart highlights the superior reliability and comprehensive performance of molecular sexing.

These results indicate that when definitive sex determination was achieved through endoscopy or necropsy, the results were fully consistent with those obtained through molecular sexing (Figure 8).

## 4 Discussion

Traditional methods for pigeon sexing rely on secondary sexual characteristics such as neck plumage, body size, and behavioral cues. Cloacal sexing is rarely used due to limited sexual dimorphism, making accurate sex determination challenging (18). In this study, we compared endoscopic, necropsy, and molecular sexing techniques in domestic pigeons in order to evaluate their accuracy, reliability, and practical applicability.

Molecular sexing emerged as the most effective method, achieving a 100% (95% CI: 86.7%−100.0%) success rate, including in juveniles with small and poorly differentiated gonads. Necropsy followed with an 84% (95% CI: 65.3%−93.6%) success rate and proved particularly valuable when endoscopic visualization was limited due to intracelomic hemorrhage, coelomitis, or adhesions. Endoscopic sexing showed the lowest efficiency (72%; 95% CI: 52.4%−85.7%), with limitations largely attributed to examiner expertise and reduced visibility in the celomic cavity.

Adult pigeons during the breeding season were easily sexed *via* endoscopy due to well-developed gonads, while juveniles posed challenges because of smaller and less differentiated gonads. Immature ovaries could be misidentified as testicles, particularly in cases with intercurrent pathologies such as intracelomic hemorrhage or coelomitis with adhesions. When visibility was compromised, necropsy provided clearer anatomical insight, though poorly differentiated gonads in juveniles sometimes resulted in inconclusive identification. Newly hatched pigeons (up to 7 days old) also presented challenges, with immature gonads leading to inconclusive results in both endoscopic and necropsy assessments (18, 23).

Among molecular sexing methods, blood clot samples were the most reliable (100%; 95% CI: 86.7%−100.0%), followed by oral swabs (88%; 95% CI: 70.0%−95.8%) and feathers (80%; 95% CI: 60.9%−91.1%). However, these results should be interpreted considering the limited sample size of the present study (*n* = 25), including only six juveniles and one newly hatched chick, which may influence the precision of performance estimates and limit generalization across age groups. Oral swabs offer a minimally invasive alternative to blood, though samples from pigeons with stomatitis lesions or pseudomembranes occasionally failed to amplify due to reduced epithelial cells and DNA quantity (20, 21, 24). Feather samples required retesting due to lower DNA quantity, though down feathers from a 7-day-old pigeon provided reliable results. These findings align with prior studies reporting the highest DNA quality from blood, followed by oral swabs and feathers (13, 25–32, 53), although larger studies are needed to confirm these differences across broader age categories.

Endoscopic sexing accuracy was limited in juveniles due to small, less differentiated gonads. Concurrent pathologies, such as intracelomic hemorrhage, coelomitis with adhesions, or egg-laying complications, further impaired visibility. Because the endoscopic examinations were performed by a novice operator, the observed 72% (95% CI: 52.4%−85.7%) success rate should be interpreted in the context of the operator's learning curve rather than as an inherent limitation of the technique itself. Initial procedures were associated with greater difficulty in anatomical orientation and gonadal identification; however, performance improved with increasing experience, particularly in cases with clear visualization, absence of pathological changes, and well-developed gonads. The examinations were performed in two separate sessions separated by several months, which may have interrupted the continuity of skill acquisition and contributed to variability in performance. Previous authors have emphasized that successful avian coelioscopy requires adequate operator experience, technical proficiency, and a thorough understanding of avian anatomy for accurate interpretation of endoscopic findings (18, 25). Although these authors recognize operator experience as a key determinant of procedural success, neither study formally evaluates the learning curve or defines the level of experience required to achieve proficiency. While the present study was not designed to assess the learning curve, noticeable improvements in procedural efficiency and confidence were observed after repeated examinations. A systematic evaluation of the learning process, similar to those reported for minimally invasive procedures in mammalian species, would be valuable for establishing training recommendations and competency in avian celioscopy. Future studies should include standardized training protocols, multiple endoscopists with different levels of experience, and larger sequential case series to better characterize the learning curve associated with endoscopic sex determination by inexperienced operators. Higher success rates reported by experienced avian clinicians further highlight the importance of training, experience, and anatomical familiarity for accurate endoscopic identification (18, 25–32).

Necropsy, while generally reliable, was affected by juvenile gonadal immaturity and pathologies such as fibrin deposits (case 4), which reduced visibility near the intestinal loops. In contrast, molecular sexing provided consistent results even in cadavers with advanced autolysis or lesions, though some samples required retesting due to reduced DNA content (15, 33–36).

Two PCR protocols were tested: P2/P8 (1) and P2/NP (22), both targeting the CHD1 gene for sex identification. Both protocols were effective in pigeons, though the P2/NP primer pair produced more distinct and easily interpretable bands and may be suitable for a broader range of species (37). Modern DNA isolation kits effectively overcome previous challenges with PCR inhibitors, such as keratin from feathers or hemoglobin from blood (33, 34). However, genetic sexing is still constrained by sample quality, feather size, feather degradation, anatomical region, oral cavity lesions, and cadaver autolysis (21, 41).

Blood remained the most reliable DNA source due to the presence of nucleated erythrocytes, providing the highest DNA concentration and purity, which translated into a 100.0% (95% CI: 86.7%−100.0%) PCR amplification success rate, although blood collection is invasive and potentially stressful (13, 20). Oral swabs produced DNA of intermediate quantity and purity and achieved successful molecular sex determination in 88.0% (95% CI: 70.0%−95.8%) of samples. As a minimally invasive sampling method, oral swabs are particularly suitable for juveniles, small birds, or individuals in which blood collection is impractical (21, 24, 38). In the present study, oral swab performance was reduced in two pigeons with suspected avian poxvirus infection, where pseudomembranous and necrotic oral lesions likely decreased epithelial cell availability and compromised DNA quality. Feather samples, especially plucked contour or flight feathers with an intact calamus, provide a minimally invasive DNA source; however, they obtained the lowest DNA concentration and purity in the present study and were associated with the lowest PCR amplification success rate (80.0%; 95% CI: 60.9%−91.1%). Molted feathers generally provide lower-quality DNA and often require larger quantities for successful amplification (24, 39–41). In addition, feather quality, size, collection method, and storage conditions have been shown to significantly influence PCR success rates (21, 24). Overall, our findings indicate that both DNA quantity and purity influence PCR amplification success and should be considered when selecting the most appropriate biological sample for molecular sex determination.

Molecular sexing offers multiple advantages, including high accuracy, speed, low cost, safety, and applicability across all age categories (6, 13, 42). Oral swabs are particularly recommended due to ease of collection, minimal handling stress, and reliable DNA quality (21, 38, 41). In the current study, two oral swab samples showed reduced DNA quality, likely due to pseudomembranes and necrotic lesions from suspected avian pox virus, which decreased epithelial cell availability and compromised PCR amplification, resulting in an overall oral swab sexing accuracy of 88% (95% CI: 70.0%−95.8%). On the other hand, oral swabs were highly effective for sexing the featherless, newly hatched pigeon, demonstrating the method's applicability even in very young birds. Blood samples provide higher DNA concentration but require careful handling to avoid stress or health risks (41). Feather and oral swab sampling enable safe, non-invasive sexing of live birds, including juveniles, without risk of infection or handling-induced stress (21, 24, 38). Even less invasive sources, such as urine (43), feces (44, 45), and regurgitated seeds (45), have been successfully used for DNA extraction and molecular sexing. These alternative sample types could further reduce handling stress, particularly in sensitive or endangered species, while still enabling accurate sex determination.

This study confirms that molecular sexing using blood, oral swabs, and feathers is the most reliable method for sex determination in domestic pigeons. Endoscopy and necropsy remain valuable, particularly in adults with well-differentiated gonads, but their accuracy is affected by gonadal immaturity, intercurrent pathologies, and examiner experience (18). The P2/NP PCR protocol is recommended for domestic pigeons, providing clear bands and high success rates (22, 37), and aligns with findings in Psittaciformes where the use of multiple markers improves reliability and avoids sex misidentification (46). Proper sample collection, storage, and handling are critical for maximizing molecular sexing reliability (13, 33, 39–41, 47).

Previous studies have demonstrated the successful use of different biological samples, including blood, buccal swabs, feathers, cloacal swabs and feces for molecular sex determination in birds (48–52). The novelty of the present study lies not in demonstrating the superiority of molecular sexing over traditional techniques, which has been well established in avian species, but in providing a comprehensive comparison of three biological sample types collected from the same domestic pigeons while simultaneously evaluating endoscopic sexing performed by a novice operator and validating the findings by necropsy. Furthermore, this study demonstrates the applicability of molecular sexing in juvenile and newly hatched pigeons, offering practical recommendations for breeders and avian clinicians regarding the most appropriate sampling strategy.

For routine sex identification, molecular sexing using oral swabs is recommended due to its reliability, minimal invasiveness, and ease of collection, particularly in juveniles and newly hatched pigeons. Blood sampling can be reserved for cases requiring the highest DNA quantity, while feather collection offers a safe alternative when handling stress must be minimized. Implementing these methods allows breeders and avian practitioners to accurately determine sex while reducing the risk of injury or unnecessary stress, thereby supporting improved management of breeding programs. Limitations of this study include the relatively small sample size, the exclusive use of deceased birds, and the lack of conventional diagnostic performance metrics (e.g., sensitivity, specificity, positive predictive value, negative predictive value, likelihood ratios, and overall accuracy) using molecular sexing as a reference standard. However, the primary aim of this study was to compare method applicability and agreement rather than to validate diagnostic performance. Additionally, the limited number of juveniles (*n* = 6) and the inclusion of only one newly hatched chick may affect the precision of performance estimates and limit generalization across age categories. The observed sex ratio imbalance (16 males: 9 females) may also reflect sampling variation rather than the characteristics of the broader population, potentially influencing the interpretation of method performance. Future studies should include larger cohorts, live birds, additional species, and alternative sample types (e.g., feces and eggshell membrane), with balanced sex ratios and broader representation of age groups, including more newly hatched chicks. Comparative evaluations across clinical, breeding, and conservation settings would further improve the accuracy and practical applicability of molecular sex determination methods.

## 5 Conclusion

Molecular sexing proved to be the most reliable approach for sex determination in domestic pigeons (*Columba livia domestica*), achieving the highest success rate (100%; 95% CI: 86.7%−100.0%) compared with necropsy (84%; 95% CI: 65.3%−93.6%) and endoscopic sexing (72%; 95% CI: 52.4%−85.7%). Molecular analysis was particularly valuable in juvenile individuals with poorly differentiated gonads, where conventional methods were limited. Among the evaluated biological samples, blood clot samples provided the highest amplification success (100%; 95% CI: 86.7%−100.0%) and the greatest DNA yield (181.68 ± 138.83 ng/μL), followed by oral swabs (88.0%; 95% CI: 70.0%−95.8%; 84.69 ± 79.85 ng/μL) and feathers. Although blood remains the most reliable DNA source, oral swabs represent a practical and minimally invasive alternative, particularly for juveniles, newly hatched pigeons, and situations where blood collection may be undesirable due to welfare considerations. Feather samples provide an additional non-invasive option but may be associated with lower DNA quantity and reduced amplification success. Overall, molecular sexing using oral swabs offers a reliable, safe, and accessible approach for routine sex determination in domestic pigeons, supporting breeding management and avian clinical practice. Further studies involving standardized training protocols, multiple endoscopists with different levels of experience, larger cohorts, live birds, balanced age groups, additional sample types, and comprehensive diagnostic evaluations, including sensitivity and specificity analyses, are required to validate these findings and further define the optimal sampling strategy across different management settings.

## Data Availability

The raw data supporting the conclusions of this article will be made available by the authors, without undue reservation.
